# Dissecting myocardial mechanics in patients with severe aortic stenosis: 2-dimensional vs 3-dimensional-speckle tracking echocardiography

**DOI:** 10.1186/s12872-020-01336-0

**Published:** 2020-01-30

**Authors:** Xiaojun Bi, Darwin F. Yeung, Husam M. Salah, Maria C. Arciniegas Calle, Jeremy J. Thaden, Lara F. Nhola, Hartzell V. Schaff, Sorin V. Pislaru, Patricia A. Pellikka, Alberto Pochettino, Kevin L. Greason, Vuyisile T. Nkomo, Hector R. Villarraga

**Affiliations:** 1 Department of Cardiovascular Diseases, Mayo Clinic, 200 First St SW, Rochester, MN 55905 USA; 2 Department of Ultrasound, Tongji Hospital, Tongji Medical College, Huazhong University of Science and Technology, Wuhan, China; 3 Faculty of Medicine, Jordan University of Science and Technology, Irbid, 22110 Jordan

**Keywords:** Aortic stenosis, Speckle-tracking echocardiography, Strain, Myocardial mechanics

## Abstract

**Background:**

Aortic stenosis (AS) causes left ventricular (LV) pressure overload, leading to adverse LV remodeling and dysfunction. Identifying early subclinical markers of LV dysfunction in patients with significant AS is critical as this could provide support for earlier intervention, which may result in improved long-term outcomes. We therefore examined the impact of severe AS and its consequent increase in LV afterload on myocardial deformation and rotational mechanics by 2-dimensional (2D) and 3-dimensional (3D) speckle-tracking echocardiography.

**Methods:**

We prospectively measured various strain parameters in 168 patients (42% female, mean age 72 ± 12 years) with severe AS and LV ejection fraction (EF) ≥50%, and compared them to normal values found in literature. 2D and 3D images were analyzed for global longitudinal strain (GLS), global circumferential strain (GCS), global radial strain (GRS), basal rotation, apical rotation, and peak systolic twist. We further assessed the degree of concordance between 2D and 3D strain, and examined their association with measures of LV preload and afterload.

**Results:**

Patients with severe AS exhibited significantly lower GLS and GRS but higher GCS, apical rotation, and twist by 2D and 3D echocardiography compared with published normal values (*P* = 0.003 for 3D twist, *P* < 0.001 for all others). Agreement between 2D- and 3D-GLS by concordance correlation coefficient was 0.49 (95% confidence interval: 0.39–0.57). GLS was correlated with valvulo-arterial impedance, a measure of LV afterload (*r* = 0.34, *p* < 0.001 and *r* = 0.23, *p* = 0.003, respectively).

**Conclusion:**

Patients with severe AS demonstrated lower-than-normal GLS and GRS but appear to compensate with higher-than-normal GCS, apical rotation, and twist in order to maintain a preserved LVEF. GLS showed a modest correlation with valvulo-arterial impedance.

## Background

Aortic stenosis (AS) is the most common native valve disease and is characterized by left ventricular (LV) pressure overload. In patients with AS, the LV faces two afterloads: one from the valvular obstruction itself and the other from reduced systemic arterial compliance [1, 2]. Progressive increases in afterload lead to LV remodeling and a change in coronary flow reserve. These alterations can cause subendocardial ischemia and fibrosis and may gradually affect LV systolic function [3, 4]. LV ejection fraction (LVEF) is the most important conventional parameter used to assess LV myocardial function. However, a decrease in LVEF usually occurs at an end stage of severe AS.

Global longitudinal strain (GLS) by 2-dimensional (2D) speckle-tracking echocardiography can be used to detect early LV systolic dysfunction and has been proposed as a more sensitive way to detect a decline in LV function in patients with AS [5–11]. However, the process of ventricular contraction is complex, as relaxation occurs in three dimensions. Therefore, 3-dimensional (3D) speckle-tracking echocardiography could provide a more precise representation of myocardial deformation [12]. New insights into LV remodeling and myocardial deformation could potentially improve our ability to identify patients with aortic stenosis who are at highest risk for adverse remodeling or early LV dysfunction [13–18]. However, few studies have comprehensively characterized myocardial deformation in patients with severe aortic stenosis by 3D speckle-tracking echocardiography [18, 19].

The objectives of this prospective study were to: 1) to determine how myocardial deformation is affected in patients with severe AS and preserved LVEF compared with normal values found in literature; 2) to compare 2D to 3D echocardiographic measures of LV myocardial deformation in patients with severe AS; and 3) to characterize the relationship between LV preload and afterload and myocardial deformation.

## Methods

### Study population

We prospectively recruited 181 patients with severe AS, defined as a mean gradient > 40 mmHg or an aortic valve area < 1.0 cm^2^, and who had a preserved LVEF, defined as ≥50%, on transthoracic echocardiography. The study was performed at Mayo Clinic, Rochester, Minnesota from November 1, 2014 to August 31, 2015. Patients were excluded if they were < 18 years of age; if they had an irregular rhythm; if they had moderate or greater aortic or mitral regurgitation; or if image quality was inadequate. The final analysis included 168 patients. This study was approved by the Mayo Clinic Institutional Review Board, and all patients gave informed consent to participate.

### Image acquisition and analysis

Each patient underwent a standard 2D and a real-time 3D transthoracic echocardiogram in the left lateral decubitus position, using commercially available equipment (IE33 and EPIQ7, Philips Medical Systems, Andover, Massachusetts) with a fully sampled matrix-array transducer (X5–1). Vital signs were measured in all patients immediately before the echocardiographic examination. Studies were performed by an experienced cardiologist (X.B.). 3D full-volume images were acquired from the apical window with a 6-beat acquisition, a high volume rate (average, ≥30 volumes/s), and full coverage of the entire LV by the pyramidal volume. Patients were instructed to hold their breath during image acquisition. Images were optimized for endocardial border visualization by adjustment of overall gain, compression, and time gain compensation before acquisitions. The acquired 2D images and 3D full-volume images were analyzed offline with TomTec 4D Echo software, version 4.6 (TomTec Imaging Systems, Image Arena, Unterschleissheim, Germany).

For 2D echocardiography, the standard 2D, M-mode, and Doppler measurements were obtained in accordance with guidelines from the American Society of Echocardiography [20]. LV end-diastolic volume (LVEDV), end-systolic volume (LVESV), and ejection fraction (LVEF) were measured manually by using the Simpson’s biplane method. Three standard apical views (4-chamber, long-axis, and 2-chamber) were obtained for the assessment of global longitudinal strain (GLS) and three parasternal short-axis views (basal, mid, and apical levels) were obtained for the assessment of global circumferential strain (GCS), global radial strain (GRS), LV apical peak systolic rotation, LV basal peak systolic rotation, and peak systolic twist.

**Fig. 1:**
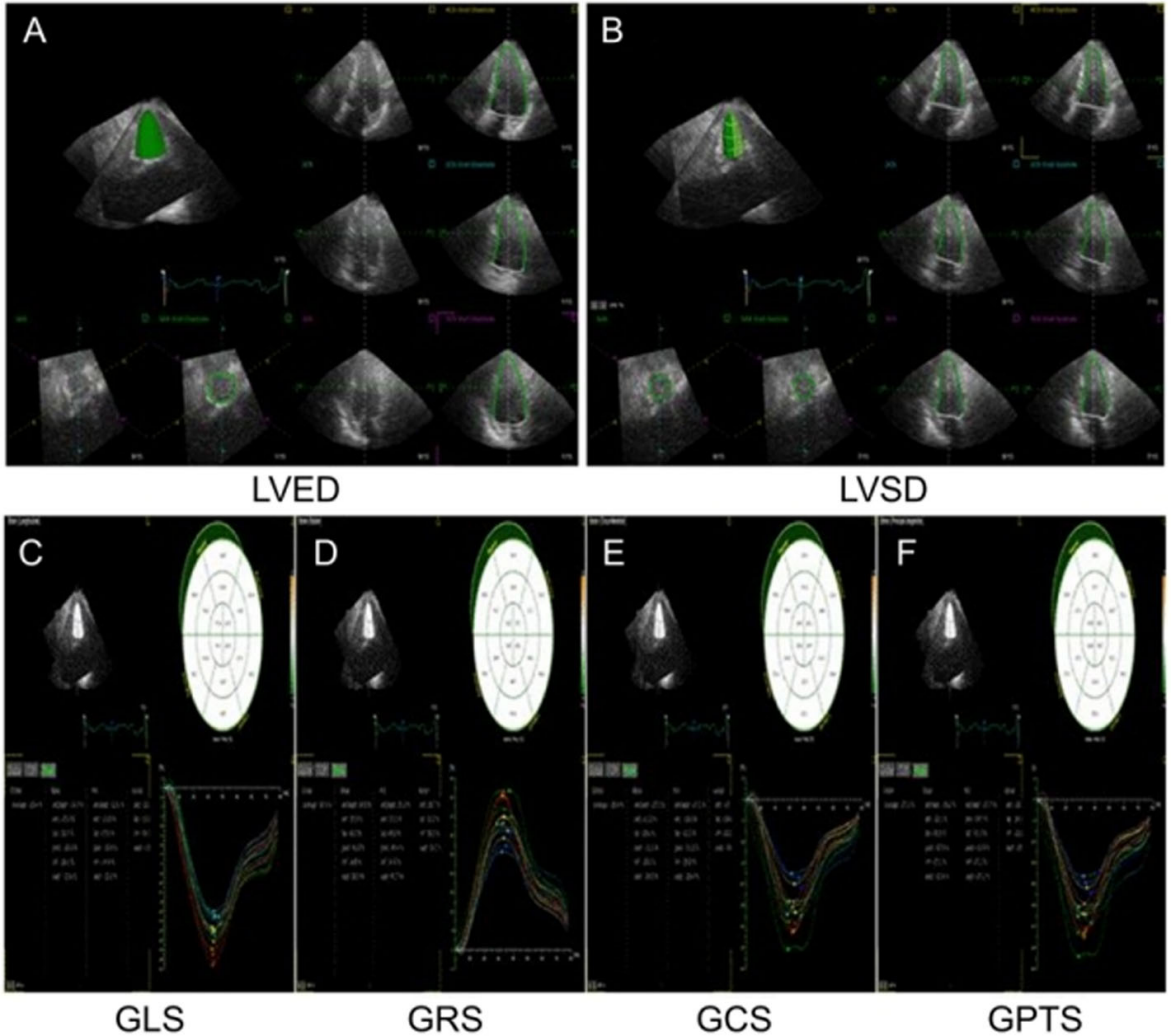
Three-dimensional (3D) speckle-tracking with TomTec 4D Echo Software (TomTec Imaging Systems, Image Arena, Unterschleissheim, Germany). Standard three apical views and one short-axis view of the left ventricle (LV) were automatically extracted from the 3D full-volume data sets. The 3D endocardial surface was automatically reconstructed at LV end-diastole (LVED) (**a**) and LV end-systole (LVSD) (**b**). The software provided global longitudinal strain (GLS) (**c**), global radial strain (GRS) (**d**), global circumferential strain (GCS) (**e**), and global principal tangential strain (GPTS) (**f**) time curves in 16 segments as well as peak global strain and averaged peak strain at three LV levels (basal, mid, and apical)

For 3D echocardiography, three standard apical views were automatically extracted from the 3D full-volume data sets. The mitral annulus and the LV apex were manually selected as the landmarks to initialize the LV boundaries. Then, the 3D endocardial surface was automatically reconstructed at end-diastole and end-systole. The endocardial surface reconstruction was manually adjusted, as necessary, and the papillary muscles were included as part of the LV cavity. Subsequently, 3D speckle-tracking was automatically characterized. The software provided longitudinal, radial, circumferential, and principal tangential strain time curves for the 16 segments and peak global strain, as well as averaged peak strain at three LV levels (basal, mid-ventricular, and apical) (Fig. 1).

To measure afterload, total arterial stiffness (TAS) was measured by the formula: TAS = pulse pressure/stroke volume (SV); total arterial compliance (TAC) was measured by the formula: TAC = SV/pulse pressure; effective arterial elastance (EAE) was measured by the formula: EAE = end systolic pressure/SV; systemic vascular resistance (SVR) was measured by the formula: SVR = [80 × (mean arterial pressure – right atrial pressure)]/cardiac output; systemic vascular resistance index (SVRI) was measured by the formula: SVRI = [80 × (mean arterial pressure – right atrial pressure)]/cardiac index [21]. Briand et al. [1] proposed a simple index to measure global LV afterload called the valvulo-arterial impedance (Z_va_), which can be calculated by the formula: Z_va_ = (SAP + MG_net_)/SVI, where SAP is the systolic arterial pressure, MG_net_ is the mean net pressure gradient transvalvular pressure, and SVI is the stroke volume index. Therefore, Z_va_ represents the valvular and arterial factors that oppose ventricular systole by absorbing the mechanical energy developed by the left ventricle [22].

### Intra- and inter-observer variability of 2D and 3D speckle-tracking measurements

For reproducibility of 2D- and 3D-speckle-tracking echocardiographic measurements of deformation parameters, 20 patients were randomly selected and reanalyzed by the same observer to determine the intra-observer agreement and by a second experienced echocardiographer, who was blinded to the initial results, to determine the inter-observer agreement. Both measurements were obtained with the intra-class correlation coefficient (ICC).

### Statistical analysis

Data were presented as mean ± standard deviation (SD) for continuous variables and as number (percentages) for categorical variables. The various strain parameters measured in this study were compared to those of normal healthy patients previously published in literature including two meta-analyses with pooled data from > 2000 patients each for both the 2D and 3D global strain measurements [23–25]. Agreement between parameters in 2D and 3D were assessed using the concordance correlation coefficient (CCC) with 95% confidence interval (CI). Associations between two continuous variables were measured using the Pearson (r) or Spearman correlation (ρ) coefficient. Variability between the two sets of measurements was reported as the mean difference ± SD and the ICC with 95% CI. Means for 2D and 3D GLS, GCS, and GRS were compared with the one-sample *t*-test using reference values obtained from two large meta-analyses [23, 24]. All other means were compared with the two-sample *t*-test. Data were analyzed with JMP 10.0 software (SAS Institute Inc., Cary, North Carolina) and MedCalc statistical software, version 11.4.1.0 (MedCalc Software, Ostend, Belgium). A *P* value < 0.05 was considered statistically significant.

## Results

### Clinical and echocardiographic characteristics

Table 1 lists the clinical characteristics of cohort patients with severe AS and preserved LVEF. A total of 168 patients were included in the study, of whom, 70 (42%) were female with a mean age of 72 ± 12 years. Hypertension and coronary artery disease were present in 80 and 49% of patients respectively.

**Table 1:** Clinical characteristics (*N* = 168)

Variable	Value
Demographics	
Age (y)	72 ± 12
Female	70 (42)
Body surface area (m^2^)	1.95 ± 0.26
Functional status	
New York Heart Association I/II/III/IV	57/57/45/9
Medical history	
Coronary artery disease	82 (49)
Diabetes mellitus	55 (33)
Hypertension	134 (80)
Dyslipidemia	113 (67)
Current tobacco use	20 (12)
Physical examination	
Heart rate (beats/min)	67 ± 12
Systolic blood pressure (mm Hg)	129 ± 18
Diastolic blood pressure (mm Hg)	70 ± 10

Data expressed as mean ± standard deviation or n (%)

Echocardiographic characteristics of cohort patients with severe AS and preserved LVEF are listed in Table 2. 2D measurements were available for all 168 patients and 3D measurements were available for 165 of them. Measures of preload in the cohort patients included an LV end-diastolic dimension of 48 ± 6 mm and an indexed LVEDV of 63 ± 20 mL/m^2^ while the calculated indices of LV afterload were as follows: total arterial stiffness, 0.8 ± 0.3 mmHg/mL; total arterial compliance, 1.5 ± 0.7 mL/mm Hg; systemic vascular resistance, 1368 ± 438 mL/mm Hg/m^2^; systemic vascular resistance index, 2628 ± 788 dynes•s/cm^− 5^; and Zva, 3.7 ± 0.7 mmHg/mL/m^2^.

**Table 2:** Echocardiographic features

Variable	Value
2D measurements (*N* = 168)	
LV end-diastolic diameter (mm)	48 ± 6
LV end-systolic diameter (mm)	30 ± 5
Interventricular septum thickness (mm)	12 ± 2
Posterior wall thickness (mm)	11 ± 2
LV mass index (g/m2)	109 ± 34
LV end-diastolic volume index (mL/m^2^)	63 ± 20
LV end-systolic volume index (mL/m^2^)	24 ± 11
LV stroke volume index (mL/m^2^)	40 ± 10
LV ejection fraction (%)	63 ± 6
E velocity (cm/s)	96 ± 42
A velocity (cm/s)	104 ± 36
E/A ratio	0.9 ± 0.4
Septal e’ velocity (cm/s)	5.4 ± 1.7
Septal E/e’ ratio	17.3 ± 7.5
Aortic valve peak velocity (m/s)	4.4 ± 0.5
Aortic valve mean gradient (mm Hg)	49 ± 12
Aortic valve area (cm^2^)	0.89 ± 0.16
Aortic valve area index (cm^2^/m^2^)	0.46 ± 0.07
Total arterial stiffness (mm Hg/mL)	0.8 ± 0.3
Total arterial compliance (mL/mm Hg)	1.5 ± 0.7
Systemic vascular resistance (mL/mm Hg/m^2^)	1368 ± 438
Systemic vascular resistance index (dynes•s/cm^−5^)	2628 ± 788
Valvulo-arterial impedance (Z_va_) (mm Hg/mL/m^2^)	3.7 ± 0.7
3D measurements (*N* = 165)	
LV end-diastolic volume index (mL/m^2^)	62 ± 16
LV end-systolic volume index (mL/m^2^)	24 ± 9
LV stroke volume index (mL/m^2^)	38 ± 9
LV ejection fraction (%)	61 ± 5

Data expressed as mean ± standard deviation

*Abbreviations*: *2D* 2-dimensional, *LV* Left ventricular, *3D* 3-dimensional

### Parameters of speckle-tracking strain imaging in 2D and 3D

The parameters of 2D and 3D speckle-tracking strain for both groups are shown in Table 3. For both 2D and 3D images, cohort patients with severe AS and preserved LVEF demonstrated lower GLS and GRS (*P*<0.0001 for all) and higher apical rotation and peak systolic twist (*P* = 0.003 for 3D twist, *P* < 0.0001 otherwise) compared with those of healthy subjects previously published in literature. There was no significant difference in 2D and 3D basal rotation in patients with severe AS compared with normal values.

**Table 3:** Myocardial mechanics

Parameter	Study cohort	Reference values	*P* value
2D measurements (*N* = 168)			
GLS (%)	−16.2 ± 2.1	−19.7 (−20.4, −18.9)^a^	< 0.0001
GCS (%)	−27.4 ± 4.6	−23.3 (−24.6, −22.1)^a^	< 0.0001
GRS (%)	37.5 ± 8.2	47.3 (43.6, 51.0)^a^	< 0.0001
Basal rotation (°)	−7.8 ± 2.2	−7.5 ± 5.4^b^	0.06
Apical rotation (°)	10.7 ± 4.0	6.3 ± 3.5^b^	< 0.0001
Twist (°)	18.5 ± 4.7	13.4 ± 8.2^b^	< 0.0001
3D measurements (*N* = 165)			
GLS (%)	−14.5 ± 1.9	− 19.1 (− 18.2, 19.9)^c^	< 0.0001
GCS (%)	−30.5 ± 7.1	−22.4 (−21.0, − 23.9)^c^	< 0.0001
GRS (%)	41.6 ± 9.8	47.5 (41.5, 53.5)^c^	< 0.0001
Twist (°)	13.7 ± 7.0	10.2 ± 7.6^b^	0.003

Data are expressed as mean ± standard deviation or mean (95% confidence interval)

*Abbreviations*: *2D* 2-dimensional, *3D* 3-dimensional, *GLS* Global longitudinal strain, *GCS* Global circumferential strain, *GRS* Global radial strain

^a^Yingchoncharoen et al. [23]

^b^Andrade et al. [24]

^c^Truong et al. [25]

### Comparison of 2D and 3D echocardiographic measurements

**Fig. 2:**
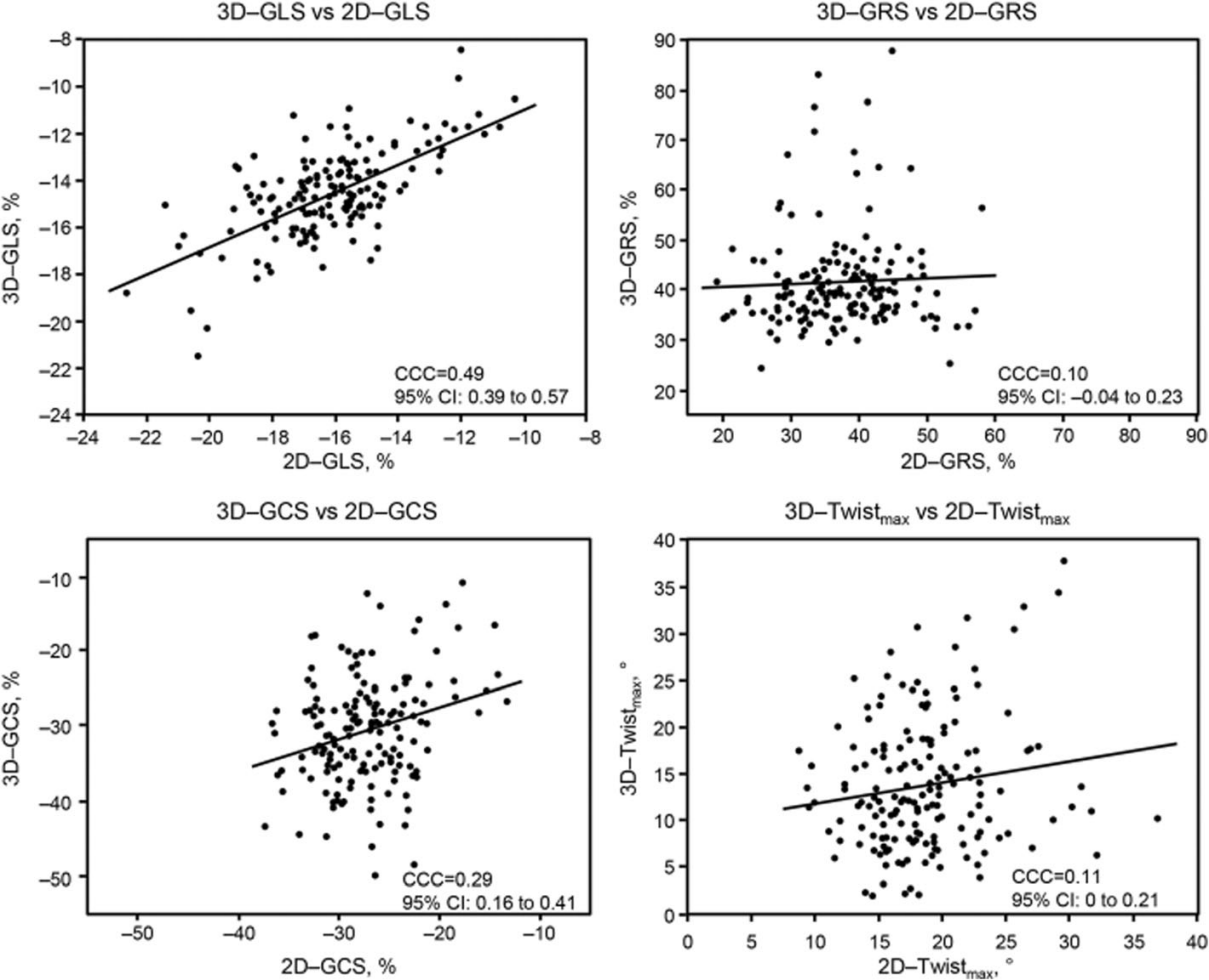
Agreement between 2-dimensional (2D) and 3-dimensional (3D) strain parameters of the left ventricle. Abbreviations: GLS, global longitudinal strain; 2D–GRS, global radial strain; GCS, global circumferential strain; Twist_max_, peak systolic twist; CCC, concordance correlation coefficient; CI, confidence interval

**Fig. 3:**
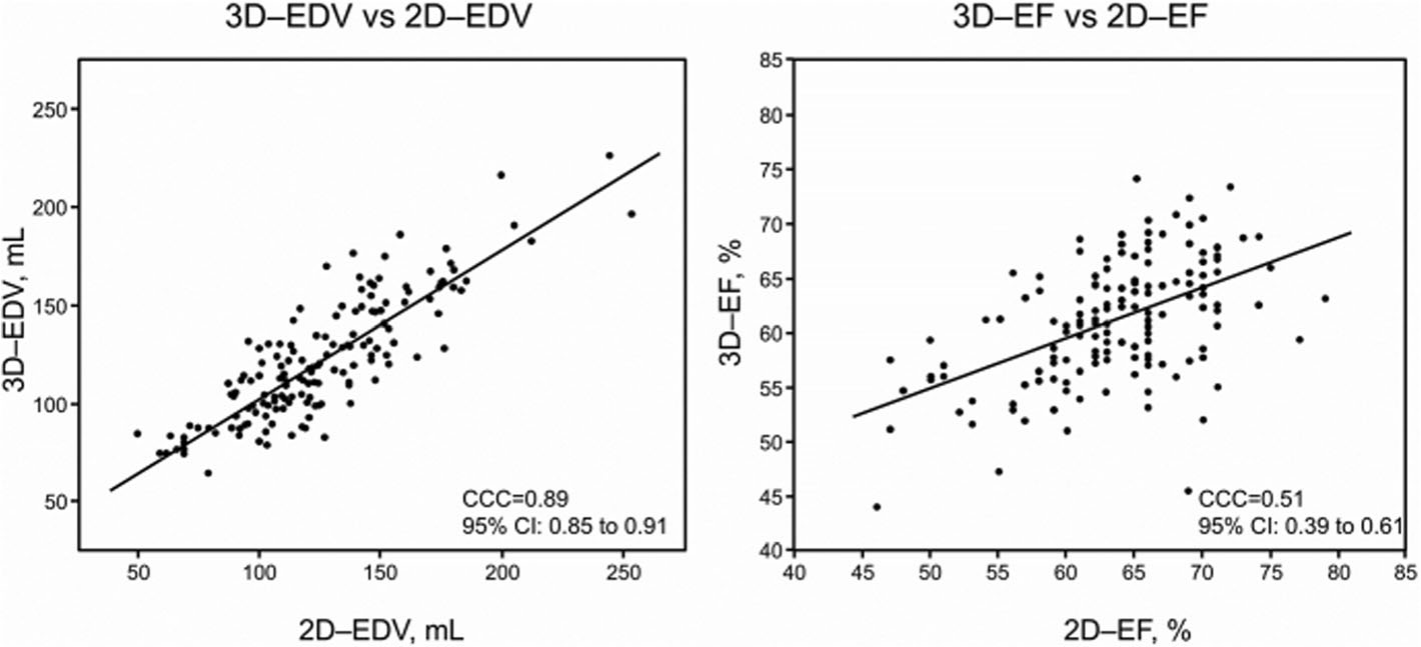
Agreement between conventional 2-dimensional (2D) and 3-dimensional (3D) echocardiographic parameters of the left ventricle. Abbreviations: EDV, end-diastolic volume; EF; ejection fraction; CCC, concordance correlation coefficient; CI, confidence interval

The agreement of echocardiographic data between 2D and 3D images is shown in Figs. 2 and 3. A relatively fair level of agreement existed between 2D and 3D GLS (CCC = 0.49, 95% CI 0.39–0.57; and *ρ* = 0.54, *P* < 0.0001) for patients with severe AS. The agreement between 2D and 3D images was poor for GCS (CCC = 0.29, 95% CI 0.16–0.41 and *ρ* = 0.23, *P* < 0.002]), and GRS (CCC = 0.10, 95% CI − 0.04-0.23 and *ρ* = − 0.2, *P* < 0.02), and peak systolic twist (CCC = 0.11, 95% CI 0–0.21). An excellent level of agreement existed between 2D and 3D LVEDV (CCC = 0.89, 95% CI 0.85–0.91) (Fig. 3). A fair level of agreement existed between 2D and 3D LVEF (CCC = 0.51, 95% CI 0.39–0.61).

### Relationship between myocardial deformation and measures of preload and afterload

GLS by 2D and 3D was correlated with Z_va_ (*r* = 0.34, *p*<0.001; *r* = 0.23, *p* = 0.003, respectively). The other deformation parameters, including apical rotation and peak systolic twist in 2D and 3D showed no correlation with Z_va_. Among all of the indexes of afterload, only Z_va_ demonstrated the modest correlation with 2D and 3D GLS. Among the parameters of preload, indexed LVEDV demonstrated a weak correlation with 2D and 3D GLS (2D GLS, *r* = 0.14, *P* = 0.04; 3D GLS, *r* = 0.22, *P* < 0.001).

### Intra- and inter-observer variability

Table 4 shows the results of the intra-observer and inter-observer variability for 2D and 3D speckle-tracking echocardiography measurements. Our results showed excellent correlation, with ICC values ranging from 0.84 to 0.95 and a mean of 0.90.

**Table 4:** Intra- and inter-observer variability (*N* = 20)

Measurement	Intra-observer		Inter-observer	
	Difference	ICC	Difference	ICC
2D				
GLS (%)	−0.08 ± 0.17	0.93	0.40 ± 0.95	0.88
GRS (%)	−0.12 ± 1.17	0.85	−0.15 ± 4.70	0.86
GCS (%)	−0.33 ± 2.59	0.92	1.01 ± 2.88	0.90
Twist (°)	0.23 ± 2.69	0.86	0.06 ± 1.92	0.93
3D				
GLS (%)	0.40 ± 0.78	0.95	−0.08 ± 1.46	0.84
GRS (%)	0.85 ± 0.89	0.87	1.38 ± 3.81	0.82
GCS (%)	0.25 ± 3.72	0.90	0.67 ± 3.48	0.89
Twist (°)	−0.07 ± 0.76	0.87	0.01 ± 0.65	0.89

*Abbreviations*: *ICC* Intraclass correlation coefficient, *2D* 2-dimensional, *GLS* Global longitudinal strain, *GCS* Global circumferential strain, *GRS* Global radial strain, *3D* 3-dimensional

## Discussion

Our study provides the most comprehensive simultaneous 2D and 3D characterization of myocardial mechanics to date in patients with severe AS and preserved LVEF. These patients demonstrated subclinical LV systolic dysfunction in the form of reduced GLS and GRS along with concomitant increases in GCS, apical rotation, and peak systolic twist, which may represent compensatory mechanisms to maintain the LVEF within normal limits. We were able to demonstrate these changes in mechanics by both 2D and 3D speckle-tracking echocardiography. Furthermore, GLS showed a weak correlation with indexed LVEDV, a measure of preload, and a modest correlation with valvulo-arterial impedance, a measure of afterload.

Characterization of myocardial mechanics through the echocardiographic assessment of myocardial deformation, or strain, can provide a more thorough representation of LV contractile function [26]. Through the measurement of strain, changes in LV myocardial contraction can be detected in three directions: longitudinal strain reflects contraction of the longitudinally arranged endocardial and epicardial fibers; circumferential strain represents contraction of the circumferentially arranged mid-layer fibers; and radial strain denotes contraction of the full-thickness LV wall. In the presence of subendocardial ischemia, longitudinal strain is generally the first to decrease, due to the longitudinal arrangement of endocardial fibers [27]. A subsequent increase in circumferential strain can compensate for declines in longitudinal strain to maintain normal radial strain and LVEF.

Patients with severe AS and preserved LVEF in our cohort exhibited lower-than-normal GLS, a finding consistent with that found in several prior studies [5–19]. In patients with severe AS, the increased afterload may lead to left ventricular hypertrophy, decreased coronary perfusion, subendocardial ischemia, and eventually myocardial fibrosis [3, 4]. The endocardium is usually the most vulnerable to increased wall stress and stress-induced ischemia with LV pressure overload, resulting in impairment in longitudinal strain before others [28]. Indeed, layer-specific strain analysis reveals a reduction in GLS limited to the subendocardial layer even in patients with mild AS, which worsened with progression to involve the other layers with increasing severity of AS [28].

Reduced GLS was accompanied by increased GCS in our cohort of patients with severe AS and preserved LVEF, in keeping with the tendency for circumferential strain to increase in order to maintain a normal LVEF [27]. However, while GLS has consistently been shown to be low in patients with severe AS, prior studies have demonstrated either a decrease [5, 10, 29] or an increase in GCS [10, 30] in these patients. Of note, patients in those previous studies with a reduced GCS tended to have a reduced LVEF whereas those with an elevated GCS tended to have a preserved LVEF. It is possible that these patients initially develop increases in GCS to counter reductions in GLS and maintain a normal LVEF but then eventually experience reductions in GCS and as a result LVEF as well.

In addition to increases in circumferential strain, apical rotation and peak systolic twist were also significantly higher than normal in our cohort of patients with severe AS and preserved LVEF, which is consistent with prior findings [7, 10, 11, 29, 31]. Prior studies have reported good correlations between LV twist derived from 2D speckle-tracking echocardiography and magnetic resonance imaging [32, 33]. The counter-coiled helical arrangement of subendocardial and subepicardial fibers generates an LV twist that has been proven to be fundamental to LV contraction and therefore LVEF [34, 35]. Similar to circumferential strain, increases in apical rotation and twist may thus also serve to compensate for the declines in GLS in order to maintain normal LVEF and cardiac output. In fact, one prior study showed that patients with lower LVEF values tended to exhibit higher degrees of apical rotation [10].

Few prior studies have assessed changes in radial strain and basal rotation in patients with severe AS [5, 7, 11]. In our cohort, patients similarly demonstrated lower GRS [5] and no difference in basal rotation [7, 11] compared with normal values. Unlike all prior studies, our study represents to our knowledge the first to characterize longitudinal, circumferential, and basal strain, along with apical rotation, basal rotation, and twist within the same cohort of patients with severe AS using both 2D and 3D speckle-tracking echocardiography.

In patients with degenerative AS, arterial compliance is frequently reduced, which contributes to increased afterload and decreased LV function. Hence, the LV is often subjected to a double afterload from valvular obstruction and from reduced systemic arterial compliance [1, 2]. Z_va_ is a simple index that provides an estimate of the afterload imposed on the LV and is an important index of AS severity and predictor of LV dysfunction and outcomes [1, 36–38]. Z_va_ is moderately elevated in patients with severe AS, and aortic valve replacement often only reduces the valvular component of afterload and has no effect on the arterial compliance of elderly patients, which may be due to other comorbidities, such as hypertension and atherosclerosis [38, 39]. Our study further demonstrated a correlation between 2D and 3D GLS and Z_va_ but no significant relationship between Z_va_ and other deformation parameters. GLS also correlated with increasing LVEDV and E/e’ ratio. The increase in LVEDV and Z_va_ represents a hemodynamic load that markedly increases wall stress and results in depressed myocardial contractility [22].

Previous studies have reported conflicting results of comparisons of 2D and 3D speckle-tracking echocardiography measurements, possibly because of major differences in the study populations such as sample size and the severity of AS, as well as methodology such as software used for measurement of strain [19, 40–43]. One prior study showed that 3D was not superior to 2D in measuring any of the three components of LV deformation [12]. Our study showed a modest agreement between 2D and 3D GLS and a poor agreement among other parameters. There was also a similar weak correlation between 2D and 3D GLS and Z_va_.

Theoretically, 3D imaging should be more accurate as it can overcome well-known limitations of 2D imaging by avoiding foreshortened apical views, providing a more complete picture of myocardial deformation in three dimensions, and reducing out-of-plane motion, which may affect the accuracy of LV strain and twist measurements. However, the lower temporal and spatial resolutions of 3D images are potential limitations that could adversely affect the accuracy of 3D measurements acquired at the lower frame rates [42, 43]. Nevertheless, our 3D measurements were still able to capture the pattern of myocardial deformation found in patients with severe AS observed on 2D, in which GLS and GRS were reduced and accompanied by an increase in GCS, apical rotation, and twist.

## Limitations

Our study had several limitations worth considering. First, it was a single-center observational study, which may reduce the generalizability of the results. However, the prospective design of the study allowed for more precise patient selection and more comprehensive data collection that would not have been possible with a retrospective design. Second, given the elderly cohort of patients with severe AS, we were unable to recruit similar healthy age- and sex-matched subjects for comparison. Nevertheless, we used, to our knowledge, the best available reference values that have been previously published in literature for comparison including two large meta-analyses of 2D and 3D global strain. Third, our patients had pertinent comorbidities such as coronary artery disease, which could theoretically influence myocardial deformation. However, it would not be possible to fully account for all comorbidities that could affect strain in these patients. Our cohort therefore reflects a real world population of patients with severe AS with comorbid conditions. Fourth, we did not validate the deformation measurements against reference standards such as tagged magnetic resonance imaging or sonomicrometry. Furthermore, the relatively low frame rate of real-time 3D echocardiographic imaging could potentially lead to underestimating strain values. As well, the relatively high body mass index in our cohort may have led to poor image quality. Nevertheless, the similar findings obtained on both 2D and 3D provided a degree of quality assurance. Finally, it may not be possible to extrapolate our exact measurements to other ultrasound machine systems or 3D speckle tracking software, although we suspect that the pattern of deformation abnormalities observed may still be consistent.

## Conclusions

In patients with severe AS, GLS and GRS are reduced while GCS, basal rotation, and twist are increased, presumably to maintain normal cardiac output and LVEF. These findings were shown by both 2D and 3D speckle-tracking echocardiography. Reductions in GLS were shown to correlate with measures of increased preload and afterload. Our study provided the most comprehensive 2D and 3D characterization of myocardial deformation to date in patients with severe AS and preserved LVEF.

## Data Availability

Data are available from the corresponding author on reasonable request.

## Abbreviations

2D: 2-dimensional
3D: 3-dimensional
AS: Aortic stenosis
CCC: Concordance correlation coefficient
E/e′: Ratio of the early mitral inflow velocity to early mitral annular tissue Doppler velocity
EAE: Effective arterial elastance
EF: Ejection fraction
GCS: Global circumferential strain
GLS: Global longitudinal strain
GRS: Global radial strain
ICC: Intraclass correlation coefficient
LV: Left ventricular
LVEDV: Left ventricular end-diastolic volume
LVEF: Left ventricular ejection fraction
MG_net_: Mean net pressure gradient
SAP: Systolic arterial pressure
SV: Stroke volume
SVI: Stroke volume index
SVR: Systemic vascular resistance
SVRI: Systemic vascular resistance index
TAC: Total arterial compliance
TAS: Total arterial stiffness
Twist_max_: Peak systolic twist
Z_va_: Valvulo-arterial impedance
